# Association of Neck Circumference with Obesity in Female College Students

**DOI:** 10.3889/oamjms.2015.118

**Published:** 2015-12-06

**Authors:** Dimitrios Papandreou, Zujaja Tul Noor, Maitha Rashed, Hadeel Al Jaberi

**Affiliations:** *Department of Natural Sciences & Public Health, Zayed University, Abu Dhabi, United Arab Emirates*

**Keywords:** asthma, Obesity, neck circumference, waist circumference, college students, United Arab Emirates

## Abstract

**BACKGROUND:**

Obesity levels have been dramatically increased in the United Arab Emirates over the last few years. High levels of body Mass Index, waist circumference, and percent of total body fat as a measure of obesity have found to be related to cardiovascular risk factors and other diseases. Neck circumference is a new tool that has been linked to obesity. However, no studies in UAE have been conducted yet.

**AIM:**

The purpose of this study was to measure the obesity levels in a college population and to correlate them with NC and other anthropometrical indexes.

**METHODS AND SUBJECTS:**

Two hundred forty three (243) female students aged 18-25 were conveniently selected to participate in the study. Anthropometrical indexes were obtained from all subjects e after fasting.

**RESULTS:**

The prevalence of overweight and obesity together was found to be 28.4 % (n = 69). Pearson correlation showed that WC, NC and BF (%) were significantly positively related to obesity, (r = 0.790; r = 0.758; r = 0.767, p < 0.001), respectively. In multiple regression analysis, only NC (Beta: 1.627, 95 %CI: 0.370, 2.846, p < 0.001) and WC (Beta: 0.464, 95 %CI: 0.135, 0.664, p < 0.001) were found to be independently associated with obesity.

**CONCLUSION:**

NC was found to be independently associated with obesity levels in Emirati college students.

## Introduction

With the global prevalence of obesity reaching epidemic proportions, an emphasis has been placed on identifying measures of obesity quickly and accurately. In North America, approximately two-thirds of the population is considered overweight, which is defined as a body mass index (BMI) ≥ 25 kg/m^2^, and of those, it is estimated that one-half are obese (BMI ≥30 kg/m^2^) [1]. Today, obesity has become one of the major contributors to the global burden of disease, increasing the risk of morbidity and mortality from cardiovascular diseases, type 2 diabetes and some cancers [2].

Obesity remains a major health issue for individuals residing in the UAE. A study conducted by Forbes ranked the UAE number 18 on a list of the world’s fattest countries, estimating 68.3% of its citizens to be overweight; making this small country one of the top regions plagued with high obesity rates. In addition, evidence from a new nationwide-based study in 2013 revealed that obesity is more prevalent in late adolescence ages, which possibly suggests its genesis from early childhood years [3]. Many simple anthropometric indices, including the body mass index (BMI), waist circumference (WC) are widely used as markers to reflect obesity or central obesity and to predict MS or other cardiovascular risks [4].

An early and quick assessment of obese individuals may improve the stratification of disease risk, as well as aid in identifying effective prevention and intervention strategies. Recently, neck circumference (NC), as a simple and time-saving anthropometric measurement, has been identified as an index of central obesity and a promising potential predictor for cardio-metabolic syndrome [5]. To our knowledge until now, no data has been published in the Gulf area on the correlation between neck circumference and obesity.

The purpose of this study is to measure the prevalence of obesity in female college students and to investigate, for the first time, the relationship of NC with obesity and other anthropometrical indexes.

## Methodology

A total of 243 female college students aged 18-25y from Zayed University participated in the study from September to December of 2014. Subjects with a history of thyroid disease, diabetes, dyslipidemia, hypertension or any other disease were excluded from the study. All measurements were taken in the morning after an overnight fasting.

Body weight was measured to the nearest (0.1 kg) wearing light clothes and without shoes using Adam MDW-250L, UK model-scale. Height was taken to the nearest (0.1 cm) using a Charder HM200P, Twaiwan, stadiometer. BMI was then calculated by dividing weight (kg) by height squared (m^2^).

Body composition was analyzed by bioelectrical impedance analysis (BIA) while blood pressure was measured by using an automated sphygmomanometer, (Riester Champion N, Germany) after 5 min of rest.

NC was measured with a regular plastic tape to the nearest 0.5 mm. The participants were standing erect with the arms hanging loosely at sides and head positioned in a horizontal straight position. The top edge of a plastic tape was placed just below the laryngeal prominence and perpendicular to the longitudinal axis of the neck, with the head positioned in the Frankfort horizontal plane. WC was measured at the midpoint between the lowest rib and the iliac crest, within 1 mm. WC was also determined at the midpoint between the lowest rib and iliac crest within 0.1 cm. All participants have signed an informed consent form and the ethics committee of Zayed University approved the study protocol.

### Statistics

The statistical package IBM SPSS Statistics 20.0 (IBM Corp.) was used for the statistical analysis of data. The Kolmogorov–Smirnov test was used in order to test the normality of distribution of values. Values were expressed as mean ± SD. For the parametric comparisons between two independent groups, a t-test was used. For the parametric correlations between independent parameters Pearson’s correlation coefficient was calculated. Finally, multiple regression analysis was performed in order to investigate possible associations of obesity to other parameters. Statistical significance was considered for *P* < 0.05.

## Results

Table 1 shows the basic characteristics of all subjects while Table 2 indicates the comparison of normal with OW/OB subjects. Sixty-nine out of 243 female college students (28.4%) were OW and OB. The OW/OB group had significantly important higher levels (p < 0.001) of NC, WC, BF (%) and FFM (%) compared to the normal group.

**Table 1:** Basic Characteristics of 243 female subjects

Age (Y)	20.55 ± 2.25
Height (cm)	157.60 ± 5.92
Weight (kg)	57.96 ± 12.92
BMI (kg/m^2^)	23.30 ± 4.86
WC (cm)	73.33 ± 10.58
NC (cm)	31.28 ± 2.40
SBP (HHmg)	112.18 ± 11.79
DBP (HHmg)	73.51 ± 10.47
BF (%)	31.38 ± 7.20
FFM (%)	68.62 ± 7.21

**Table 2:** Anthropometric characteristics of obese/overweight and normal female subjects

	Normal (n=173)	OW/OB (n=69)	P value
Age (Y)	20.34 ± 2.02	21.09 ± 2.69	0.195
Height (cm)	157.70 ± 5.95	157.37 ± 5.87	0.691
Weight (kg)	51.75 ± 6.22	73.53 ± 12.24	0.001*
BMI (kg/m^2^)	20.79 ± 2.02	29.61 ± 4.12	0.001*
WC (cm)	68.97 ± 7.32	84.25 ± 9.57	0.001*
NC (cm)	30.72 ± 1.93	32.68 ± 2.88	0.001*
SBP (HHmg)	111.50 ± 10.48	113.88 ± 14.52	0.156
DBP (HHmg)	72.99 ± 10.03	74.81 ± 11.46	0.224
BF (%)	28.70 ± 5.78	38.09 ± 5.97	0.001*
FFM (%)	71.30 ± 5.79	61.91 ± 5.98	0.001*

* Statistically significantly difference at P < 0.005, Data presented as mean ± SD Abrev: BMI: Body Mass Index, WC : Waist Circumference, NC: Neck Circumference; SBP: Systolic Blood Pressure, DBP: Diastolic Blood Pressure, BF: Body Fat; FFM: Free Fatty Mass.

Pearson correlation showed that NC, WC and BF (%) were strongly positively correlated with obesity (Table 3).

**Table 3:** Person correlation of obesity with other variables

	r	P
Age (y)	0.165	0.012*
WC (cm)	0.790	0.001*
NC (cm)	0.758	0.001*
BF (%)	0.767	0.001*

* Statistically significantly difference at P<0.05. Abrev: WC: waist circumference, NC: neck circumference, BF: body fat levels

Table 4 presents the correlation of NC with all the other variables. NC was found to be moderately significantly correlated with BMI (r = 0.521), WC (r = 0.453) and BF% (r = 0.478), respectively.

**Table 4:** Pearson correlation of NC with other variables

	r	P
Height (cm)	0.263	0.001*
BMI (kg/m^2^)	0.521	0.001*
WC (cm)	0.453	0.001*
BF (%)	0.478	0.001*

* Statistically significantly difference at P<0.05. Abrev: BMI: body mass index, waist circumference, NC: neck circumference, BF: body fat levels.

In Multiple regression analysis (Table 5) only NC (Beta: 1.627, 95 %CI: 0.370, 2.846, p < 0.001) and WC (Beta: 0.464, 95 %CI: 0.135, 0.664, p < 0.001) were found to be independently associated with obesity.

**Table 5:** Multiple regression analysis of different variables with obesity

Variable	Beta	95% CI	P
Age (y)	0.002	-0.185, 0.192	0.965
WC (cm)	0.464	0.135, 0.664	0.001*
NC (cm)	1.627	0.370, 2.846	0.001*
BF (%)	0.520	0.256, 0.462	0.086

* Statistically significantly difference at P<0.05. Abrev: WC: waist circumference, NC: neck circumference.

## Discussion

This is the first time in UAE and Gulf countries to present data on NC and its relation to other anthropometrical indices. In our study, sixty-nine out of 243 female college students (28.4%) were OW and OB. This prevalence is much lower than what it has been observed in other Gulf countries such as Kuwait (135%) [6], Bahrain (79.7%) [7], Qatar (32%) and UAE (44%) [8, 9]. Traditional and cultural restrictions in lifestyle of women in Gulf countries are one main reason for increased rates of obesity among women. Females have limited access to sports and exercise activities. Besides, there is an easy availability of cheap migrant labor for household work. Other reasons may include, excessive use of Internet and watching TV as well as an increased consumption of fast food in malls [9].

In our study, WC was found to be strongly positively correlated and independently associated with obesity. It has been established that abdominal obesity, assessed by WC, predicts obesity-related health risk [10], and the weighted evidence indicates that WC coupled with BMI predicts health risk better than does BMI does alone. In fact, recent findings indicate that WC is a stronger marker of health risk than BMI [11]. Moreover, WC is a component of metabolic syndrome and a predictor of development of Diabetes type 2 which is the leading cause of deaths in four 4 Gulf countries (Kuwait, Oman, United Arab Emirates and Saudi Arabia) [12].

The main finding of this study is the strong correlation observed between NC and BMI, WC and BF% levels. In addition, NC was independently associated with obesity. NC together with WC and BF levels may play a major positive role in the development of future health problems of these patients, thus it is mandatory that preventive actions should be taken in consideration seriously. NC has very recently been found to be independently associated with Non Alcoholic Fatty Liver Disease [13], metabolic syndrome [14], and cardiovascular disease and stroke in adult populations [15].

Finally, in our study total body fat was also strongly related to obesity. BIA has been found to be a valid method for assessing body composition of overweight/obese women [16] and associated with early atherosclerosis [17].

This study has a lot of limitation since we data was not collected on dietary habits, physical activity, socioeconomic status, blood lipids and genetics. Nevertheless, this study presents data, for the first time, on the relationship between NC and obesity and other anthropometric indices.

In conclusion, neck circumference is a new tool that may be used safely to measure neck fatness levels. In addition, NC was found to be independently associated with obesity levels in Emirati college students. More extended studies including data from all Emirates are necessary for the development of cut off references and the possible associations with CV risk factors that may lead to health problems for obese people later in their life.
